# Aluminum plasmonic waveguides co-integrated with Si_3_N_4_ photonics using CMOS processes

**DOI:** 10.1038/s41598-018-31736-4

**Published:** 2018-09-06

**Authors:** George Dabos, Athanasios Manolis, Dimitris Tsiokos, Dimitra Ketzaki, Evangelia Chatzianagnostou, Laurent Markey, Dmitrii Rusakov, Jean-Claude Weeber, Alain Dereux, Anna-Lena Giesecke, Caroline Porschatis, Thorsten Wahlbrink, Bartos Chmielak, Nikos Pleros

**Affiliations:** 1 Department of Informatics, Center for Interdisciplinary Research and Innovation, Aristotle University of Thessaloniki, 10th Km Thessalonikis-Thermis Av., 57001 Thessaloniki, Greece; 2 Laboratoire Interdisciplinaire Carnot de Bourgogne, UMR 6303 CNRS-Université de Bourgogne, de Bourgogne, France; 3 AMO GmbH, Advanced Microelectronic Center Aachen (AMICA), Otto-Blumenthal-Strasse 25, 52074 Aachen, Germany

## Abstract

Co-integrating CMOS plasmonics and photonics became the *“sweet spot”* to hit in order to combine their benefits and allow for volume manufacturing of plasmo-photonic integrated circuits. Plasmonics can naturally interface photonics with electronics while offering strong mode confinement, enabling in this way on-chip data interconnects when tailored to single-mode waveguides, as well as high-sensitivity biosensors when exposing Surface-Plasmon-Polariton (SPP) modes in aqueous environment. Their synergy with low-loss photonics can tolerate the high plasmonic propagation losses in interconnect applications, offering at the same time a powerful portfolio of passive photonic functions towards avoiding the use of bulk optics for SPP excitation and facilitating compact biosensor setups. The co-integration roadmap has to proceed, however, over the utilization of fully CMOS compatible material platforms and manufacturing processes in order to allow for a practical deployment route. Herein, we demonstrate for the first time Aluminum plasmonic waveguides co-integrated with Si_3_N_4_ photonics using CMOS manufacturing processes. We validate the data carrying credentials of CMOS plasmonics with 25 Gb/s data traffic and we confirm successful plasmonic propagation in both air and water-cladded waveguide configurations. This platform can potentially fuel the deployment of co-integrated plasmonic and photonic structures using CMOS processes for biosensing and on-chip interconnect applications.

## Introduction

The roadmap towards cost-effective and practical plasmonic waveguide circuits has been well identified to proceed along the use of CMOS compatible and CMOS manufacturable metals jointly with the co-integration of plasmonics with a low-loss photonic platform. Demarcating from noble metals to CMOS compatible metals that can be deployed as plasmonic waveguides within a CMOS foundry will allow for low-cost and volume manufacturing plasmonic circuitry, which can be seamlessly incorporated into the constantly growing portfolio of CMOS photonics. At the same time, co-integrating plasmonic waveguides with a low-loss photonic platform emerges as the prominent paradigm towards coping with the high propagation losses of plasmonics, facilitating the employment of plasmonics only in circuitry segments where their functional benefits are unmatched, as has been already identified in the case of biosensing and data transmission applications^1–9^.

The quest for CMOS compatible metals suitable for replacing noble-based plasmonic counterparts has, so far, been focused on materials like aluminum (Al), copper (Cu) and titanium-nitride (TiN). While promising results have been reported for all three material platforms with respect to their plasmonic properties, there are still several challenges to be addressed, associated either with their stringent manufacturing requirements or their suitability in view of certain applications or even their compatibility with CMOS processes, thereby limiting their perspectives for their practical deployment by CMOS foundries. More specifically, copper based plasmonic waveguides can provide plasmonic propagation lengths (L_spp_, defined as the 1/e decay of the power) similar to that obtained when using noble metals at 1.55 μm wavelength range^10,11^, but the oxidation of Cu necessitates in most cases the deposition of additional protective layers through further post processing steps to allow, for example, its potential exploitation in biosensing applications^12^. On the other hand, TiN-based plasmonic counterparts have demonstrated a strong dependence of their performance to the applied manufacturing conditions and the substrate material platform^13^, resulting also in L_spp_ lengths that can be much lower than those obtained by using noble metals when strong mode confinement is targeted^10^. Finally, Al appears as a widely accepted CMOS compatible material with prompted credentials towards the realization of functional plasmonic waveguides, benefiting also from its native alumina layer that is formed directly upon its exposure to ambient conditions but stabilizes at a small thickness of 2–3 nm^14^, serving then as an inherent protective layer. As such, Al holds the promise of being widely adopted in several applications with operating wavelengths spanning from visible to infrared regime. However, its SPP waveguiding characteristics at 1550 nm spectral window have been only marginally addressed so far. Only a very limited number of Al-based plasmonic waveguide implementations at 1.55 μm has been reported, having either pure L_spp_ lengths^10,15^ or multimodal waveguide properties^16^. In addition, they have never been evaluated with respect to their signal integrity characteristics, hence limiting their potential for practical employment in on-chip data transmission applications. At the same time, the highest L_spp_ of 29 μm^10^ for single-mode Al-based waveguides has been demonstrated by means of Dielectric-Loaded SPP waveguides that are inherently contradicting the SPP mode exposure to liquid-based claddings. Consequently, Al-based plasmonic waveguide characteristics in liquid environment remain still an unknown parameter, preventing in this way the substitution of noble-based plasmonic waveguides by CMOS Al based counterparts in SPP biosensors. Finally, single-mode Al SPP waveguides have never been, so far, deployed as co-integrated elements on a low-loss photonic waveguide platform offering at same time plasmonic propagation lengths higher than a few micrometers.

In this work, we demonstrate, for the first time to our knowledge, a plasmo-photonic platform manufactured entirely by CMOS processes and employing single-mode Al plasmonic stripes successfully co-integrated with a low-loss Si_3_N_4-_based photonic waveguide platform, reporting the highest L_spp_ among all single-mode Al-based plasmonic waveguide structures presented so far at 1550 nm^10,15^. Based on the outcomes of the study reported in^10^, we selected Al as the plasmonic material of choice versus other candidate CMOS metals like copper and titanium nitride. In particular, Al offers a good balance between propagation losses and durability to liquid claddings holding promise for both on-chip interconnects and biosensing applications. In this context, and to demonstrate a broader potential impact of the reported technology, the transmission performance of the Al-based plasmonic stripe waveguides has been evaluated both in data-carrying conditions with 25 Gb/s optical data signals for potential use in on-chip data transmission applications as well as in aqueous environment in view of its future employment in biochemical sensing configurations. The proposed co-integration scheme exploits the optical functions provided by the low-loss Si_3_N_4_ platform, facilitating fiber-to-chip coupling through grating couplers^17^ and allowing the on-chip excitation of SPP modes through a butt-coupled Al-to-Si_3_N_4_ interface, consequently negating the utilization of bulk optics for the excitation of SPP modes on-chip. Experimental measurements revealed a minimum plasmonic propagation loss of 0.068 dB/μm in air (L_spp-air_ = 63.8 μm) and 0.087 dB/μm in water (L_spp-water_ = 50 μm) at 1.55 μm. A minimum Al-to-Si_3_N_4_ interface loss of 4.4 and 2.8 dB at 1.55 μm has been experimentally measured in air and water, respectively. The signal integrity credentials of the Al-based plasmonic waveguides have been verified by transmitting modulated optical data at 25 Gb/s over an Al SPP waveguide with a length of 200 μm, revealing error-free operation with 0.3 dB additional power penalty.

**Figure 1:**
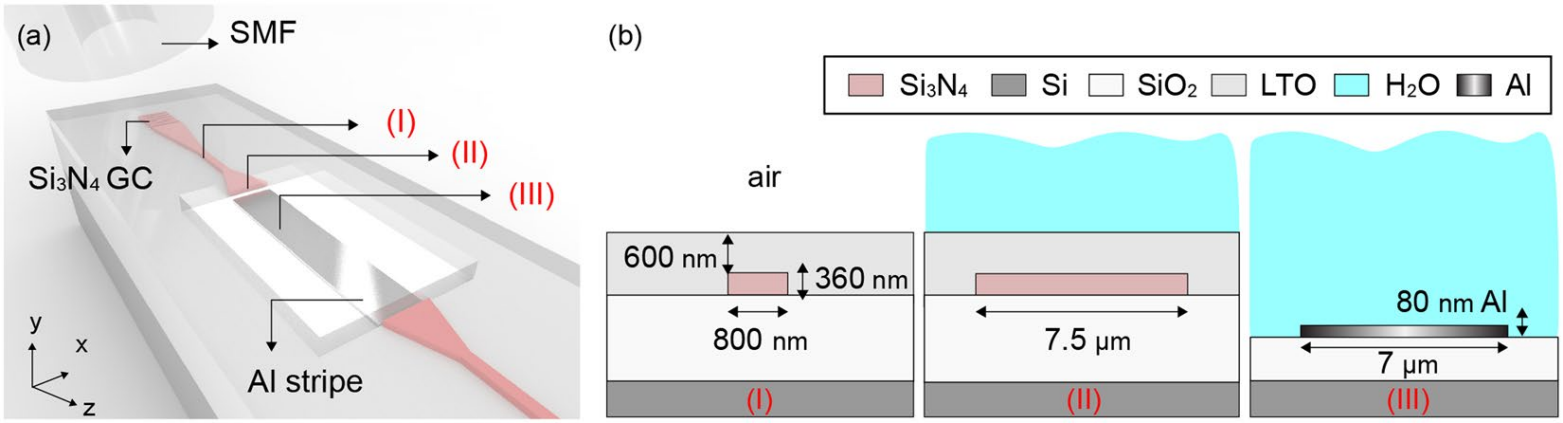
Al plasmonic stripes co-integrated with Si_3_N_4_ photonics. (**a**) 3D schematic in perspective view illustrating the proposed co-integration scheme, where Al stripes have been interface with Si_3_N_4_ waveguides exploiting a butt-coupled Al-to-Si_3_N_4_ interface and grating couplers (GCs) for fiber-to-chip coupling. The Al stripe is deposited between the photonic waveguides after fully etching Low-Temperature-Oxide (LTO), Si_3_N_4_ and partially the SiO_2_ layers. (**b**) Dimensions of the photonic and plasmonic waveguide cross-sections. Considering a liquid based top cladding material, water covers fully the plasmonic waveguide and the tapered photonic waveguide partially at its junction with the plasmonic waveguide facet.

The proposed co-integrated CMOS plasmo-photonic platform is illustrated in Fig. 1(a), where Al plasmonic stripes with dimensions of 80 nm × 7 μm (height × width) were deposited between the Si_3_N_4_ waveguides, exploiting a butt-coupled Al-to-Si_3_N_4_ interface and Si_3_N_4_ based Grating-Couplers (GCs) to facilitate fiber-to-chip coupling. The Si_3_N_4_ waveguide dimensions relied on the waveguide technology readily available by the foundry. Subsequently, the plasmonic stripe waveguide and its interface with the photonic waveguide was designed and optimized with the aid of a photonic taper and a cladding cavity as described in Fig. 1. In addition, a 7 μm wide Al based SPP waveguide has been selected to maintain single mode-condition and low plasmonic propagation losses, allowing the same time for compact layouts in view of their future employment in multichannel integrated sensor configurations. More specifically, light coming from a Single-Mode-Fiber (SMF), aligned to the Transverse-Magnetic (TM) polarization, is coupled to the 360 nm thick and 800 nm wide Si_3_N_4_ waveguide. Linear photonic tapers have been employed to facilitate modal matching between the modes supported by the photonic and the SPP waveguides. Figure 1(b) depicts the waveguide cross-sections along with their structural dimensions for the employed photonic and plasmonic waveguides.The butt-coupled Al-to-Si_3_N_4_ interface has been designed using a 3D Finite-Difference-Time-Domain (FDTD) method^18^ and was optimized for water-cladded plasmonic waveguide stripes in view of its future employment also in biochemical sensing configurations, where mostly water-based analytes are used.

**Figure 2:**
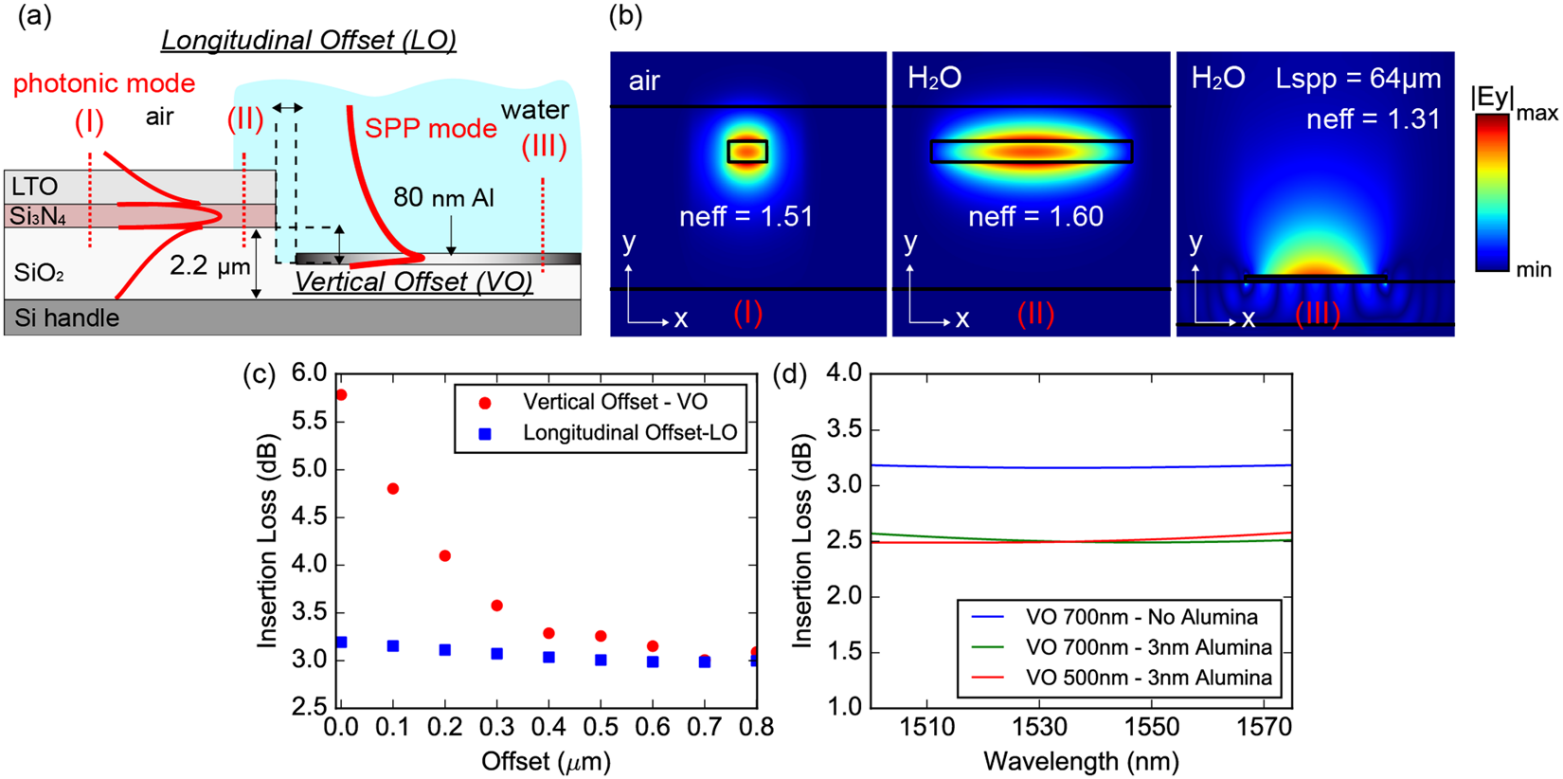
The butt-coupled Al-to-Si_3_N_4_ interface. (**a**) Side-view of the proposed interface. (**b**) Quasi-TM mode profiles (|E_y_|) supported by the different waveguide cross-sections. (**c**) Simulated Al-to-Si_3_N_4_ insertion loss, per transition, for varied LO and VO values at 1.55 μm, without considering an alumina layer. (**d**) Simulated Al-to-Si_3_N_4_ insertion loss, per transition, versus wavelength for VO of 500 nm, considering the formation of an alumina layer with a thickness of 3 nm and for VO of 700 nm with, as well as, without considering an alumina layer. The LO value during simulations was kept fixed at 500 nm.

Figure 2(a) illustrates a side-view impression of the proposed interface, where water covered fully the plasmonic waveguide and partially the photonic waveguide at the tapering section. Vertical and longitudinal offsets (VO and LO) between the waveguide facets have been applied to further reduce the insertion loss of the Al-to-Si_3_N_4_ interface by maximizing the overlap integral between the plasmonic and the photonic mode. The quasi-TM mode profiles (|Ey|) for the Si_3_N_4_ strip (I), tapered (II) and SPP (III) waveguides are depicted in Fig. 2(b,c) presents the Al-to-Si_3_N_4_ insertion loss, per transition, for different values of LO and VO at 1.55 μm, without considering the formation of native alumina layer atop the Al stripes. Simulations dictated an optimal VO offset of 700 nm resulting in an insertion loss of 3.3 dB at 1.55 μm. Based on simulation results, LO values being in the range of 0 up to 800 nm, yielded an insertion loss of about 3–3.5 dB, hence suggesting high immunity to fabrication errors. Similar tolerance is also predicted by simulations regarding the optimal VO value, showing less than 0.5 dB increase to the insertion loss for deviations of 100 nm or more around the optimal value. Figure 2(d) shows the Al-to-Si_3_N_4_ insertion loss versus wavelength for VO of 700 nm as well as 500 nm, with and without considering the formation of the native alumina layer. Simulations have shown that the presence of a 3 nm thick alumina layer can lead to a slight improvement of the interface insertion loss, owing probably to the increased effective index of the SPP mode. The simulation results when considering a VO of 500 nm correspond to the VO value adopted in the fabricated structures. More details regarding the conducted numerical simulations are provided in the methods section.

## Results

The Si_3_N_4_ building blocks including GCs, the plasmonic cavity and all the Si_3_N_4_ waveguides have been fabricated using optical projection lithography. E-beam lithography has been used for the fabrication of Al plasmonic stripes in the preparation of the first samples in order to ensure high lithographic resolution and high accuracy in critical dimensions of plasmonic stripes, prior proceeding to the transfer of the entire fabrication process in the same CMOS foundry where optical lithography was used for both photonic and plasmonic structures in the preparation of a second run of samples. The detailed fabrication process flow is presented in the methods section and accompanying schematics are provided in the Supplementary Figs S2–S4.

### Optical characterization of Al plasmonic stripes in air and data transmission experiment

**Figure 3:**
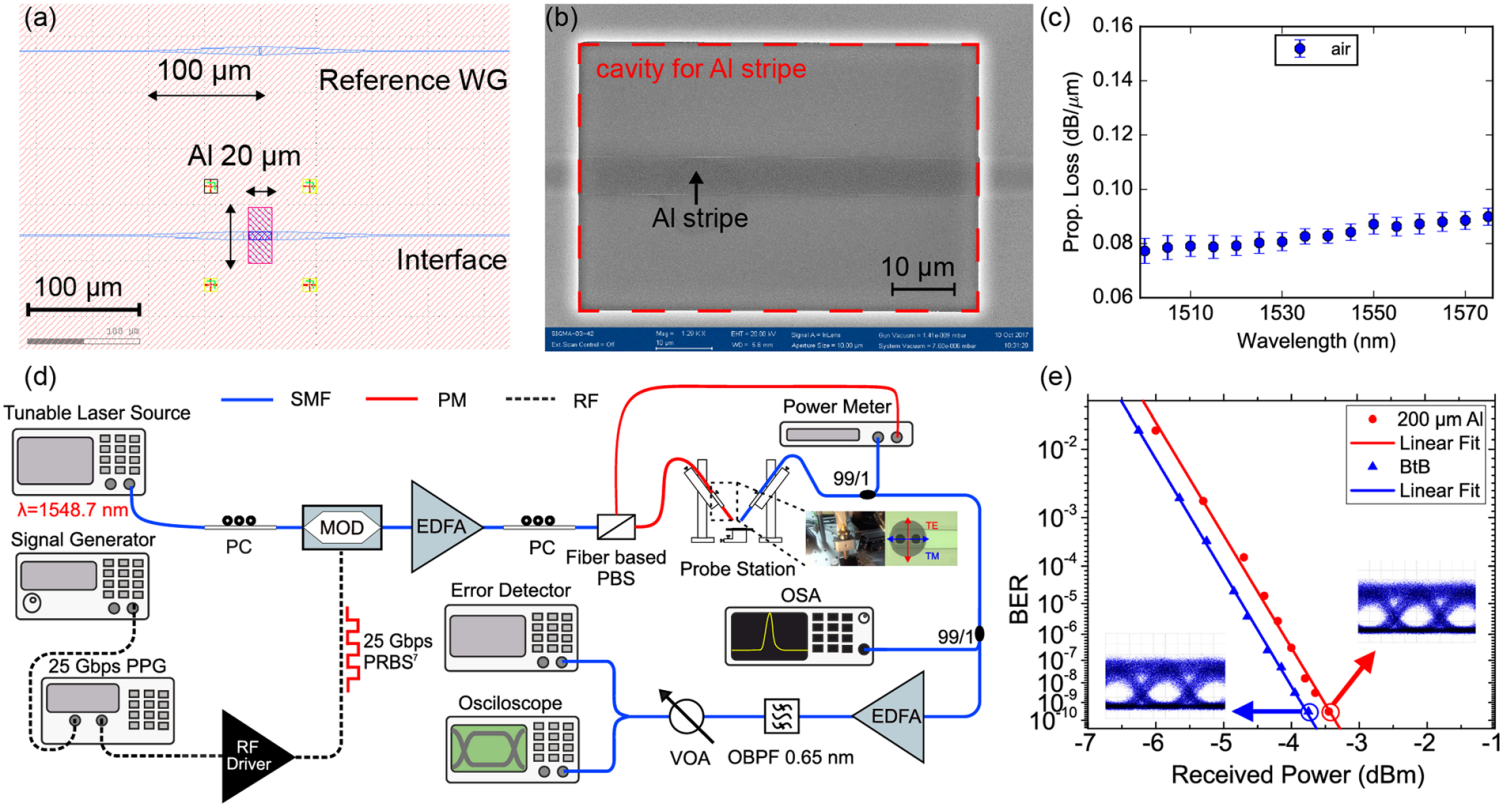
Characterized Al plasmonic stripes in air as a data carrying medium. (**a**) Mask layout depicting the reference and the Al-to-Si_3_N_4_ interface test structures used for the evaluation of Al plasmonic stripes. (**b**) SEM image illustrating in a top-view an SPP stripe with a length of 70 μm. (**c**) Propagation loss of the plasmonic mode as function of wavelength in air. The error bars refer to the standard deviation obtained from the linear fitting process. (**d**) The experimental setup that has been utilized during the 25 Gb/s transmission experiment and (**e**) the obtained eye diagrams and BER curves.

In a first step we evaluated the Al plasmonic stripes being exposed to air, by performing broadband cut-back measurements through a Fiber-to-Fiber (FtF) based setup. Figure 3(a) illustrates a part of the employed test structures in the form of a mask layout. More specifically, Al-to-Si_3_N_4_ test structures incorporated plasmonic stripes with varied length starting from 20 to 250 μm, while a reference waveguide structure has been utilized to allow calibration of the measurements. Although the longer structures accumulate significant propagation losses, they are still used in this work as the means to evaluate the signal transmission capabilities in longer interconnection scenarios while evaluating accurately the propagation losses. However, in the context of on-chip interconnects it would be more realistic to deploy shorter structures yielding lower losses, while deploying DLSPP waveguides in interconnections above 100 μm. A Si_3_N_4_ waveguide with a length of 1 cm incorporating two linear tapers with a length of 100 μm, that have been interconnected to each other with an additional photonic waveguide, has been used to form the reference waveguide structure. The additional photonic waveguide that has been used to connect the two linear tapers in the middle of the reference waveguide had a length of 100 μm and a width of 7.5 μm. Figure 3(b) depicts a Scanning-Electron-Microscope (SEM) image of an SPP waveguide with a length of 70 μm being deposited between the photonic waveguides. Figure 3(c) shows the propagation losses of the plasmonic mode in air as a function of wavelength. Measurements have shown a plasmonic propagation loss of 0.087 dB/μm in air (L_spp-air_: 50 μm) at 1.55 μm and quite broadband operation. The numerically simulated L_spp_ length at 1.55 μm is 104 μm considering an ideal plasmonic surface without roughness and possibly slightly different material properties from the actually fabricated structure. An Al-to-Si_3_N_4_ interface insertion loss of 4.4 dB at 1.55 μm in air, per transition, has been also experimentally measured. Measurement results referring to the FtF loss values as a function of wavelength for the characterized structures is shown in the Supplementary Fig. S5.

After evaluating the Al plasmonic propagation loss over wavelength, we transmitted modulated optical data at 25 Gb/s over a 200 μm long Al SPP waveguide. The main purpose of this experiment was to evaluate the quality of Al plasmonic stripes through Bit-Error-Rate (BER) measurements, hence providing insight on their signal integrity characteristics as well as experimental evidence on the fabrication readiness of Al plasmonic waveguides for possible future realization of plasmo-photonic data carrying on-chip transmission links at telecom wavelengths. The experimental setup that has been utilized during the 25 Gb/s data transmission experiment is shown in Fig. 3(d). A Continuous-Wave (CW) signal from a Tunable-Laser-Source (TLS) was tuned at 1548.7 nm and launched into a Mach-Zehnder-Interferometer (MZI) modulator based on LiNbO_3_ through a SM fiber. The MZI modulator was driven by a Pulse-Pattern-Generator (PPG), that was clocked by a signal generator at 25 Gb/s, with a 2^7-1^ non-return-to-zero (NRZ) Pseudorandom-Binary-Sequence (PRBS^7^) of electrical data. An Erbium-Doped-Fiber-Amplifier (EDFA) has been used to amplify the optical signal, yielding an optical signal at the output with 28 dBm of optical power. TM polarized light was fed into the chip using a polarization controller and a fiber based Polarization-Beam-Splitter (PBS) with Polarization-Maintaining-Fiber (PMF) pigtails. The total loss exhibited by the transmission link was 53 dB at 1548.7 nm, which is attributed to the insertion loss of: two Al-to-Si_3_N_4_ interfaces (9 ± 0.5 dB), two Si_3_N_4_ GCs (26 dB), the plasmonic propagation loss of a 200 μm long Al SPP stripe (17 ± 0.2 dB) and the propagation loss of the Si_3_N_4_ waveguide with a length of 0.88 cm (0.5 dB). Therefore, a second EDFA was employed to amplify the weak output signal providing 18 dBm of total output power, in conjunction with an Optical Band-Pass Filter (OBPF - 0.65 nm bandwidth) filtering out the noise that was introduced to signal by the EDFA. Finally, the optical signal is injected to an error detector and an oscilloscope. Figure 3(e) illustrates the obtained BER curves, showing error free operation with a maximum power penalty of 0.3 dB. Although the results show a good performance of Al-based waveguides longer than 200 μm, in the application context of on-chip interconnects it would be more realistic to deploy shorter structures coming with lower losses, as has been already applied in the case of thermo-optic plasmonic switches^7–9^ and electro-optic plasmonic modulator configurations^5,6^.

### Optical characterization of Al plasmonic stripes in water

**Figure 4:**
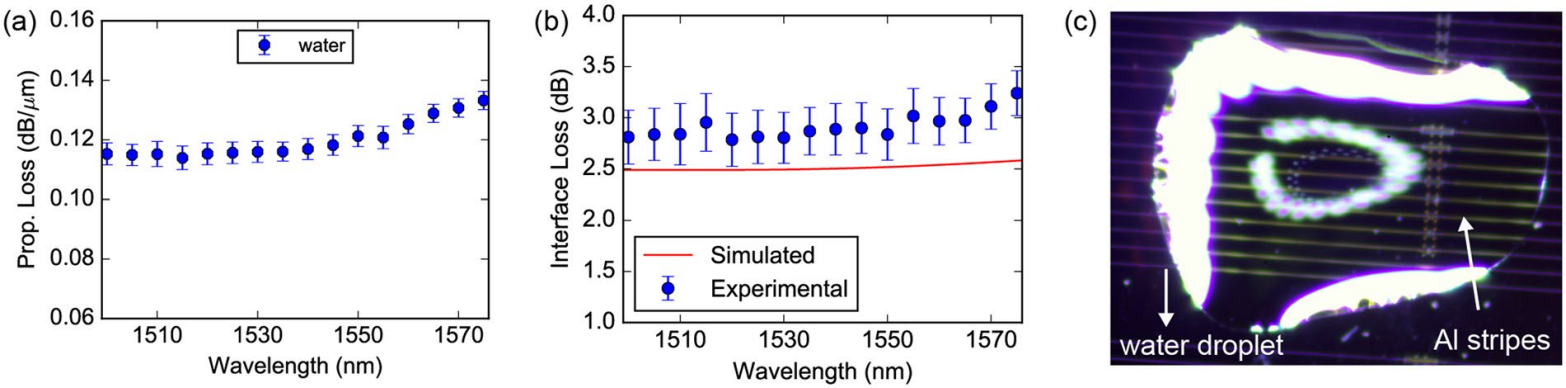
Al plasmonic stripes with propagating SPP modes in water. (**a**) Propagation loss of the plasmonic mode as function of wavelength in water. (**b**) Comparison between the experimental and the simulated Al-to-Si_3_N_4_ interface loss, per transition, as a function of wavelength in water. The simulated interface loss takes into account a VO of 500 nm and an aluminum layer with a thickness of 3 nm lying on top of the SPP stripe. The error bars refer to the standard deviation obtained from the linear fitting process. (**c**) Microscope image depicting a drop of water atop the plasmonic cut-back section.

Following the optical characterization when having air on top of the Al waveguides, a drop of water atop the plasmonic waveguides was injected to allow for the performance evaluation of the plasmo-photonic waveguide platform when the plasmonic section is exposed to aqueous environment. Figure 4(a) illustrates the experimentally measured plasmonic propagation losses over wavelength in water. Measurements revealed propagation losses of 0.121 dB/μm for the SPP mode in water resulting in an L_spp-water_ of 35.8 μm at 1.55 μm. The numerically simulated L_spp_ in case of water was 64 μm at 1.55 μm. Mismatches between experimental and numerically simulated values could be potentially attributed to fabrication imperfections and slightly inaccurate material properties of Al considered in our simulations. Compared to the case of air-cladded plasmonic propagation over Al stripes, the plasmonic propagation losses were higher in the aqueous environment due to the increased confinement of the SPP mode on the Al surface that in turn originates from the higher refractive index of water compared to that of air. Figure 4(b) illustrates the experimentally measured insertion losses of the Al-to-Si_3_N_4_ interface over wavelength in case of water cladding. An insertion loss of 2.8 dB at 1.55 μm was experimentally measured being in close agreement with the 2.5 dB losses predicted by simulations, when a 3 nm thick alumina layer is taken into account for LO and VO values equaling to the respective values used in the fabricated samples. Figure 4(c) shows a drop of water that was injected atop a plasmonic cut-back section.

Following the good agreement between numerical and experimental results, we calculated the accumulated phase change per refractive index unit (RIU) over a propagation distance equal to one micron and an L_spp_ of 104 μm, using the commercial-grade simulator eigenmode solver of *Lumerical Solutions* software. A refractive index change of Δn = 0.01 was introduced to the overlying water and the numerical results dictated an SPP mode with an effective index (n_eff_) of 1.322, increased by Δn_eff_ = 0.0097 compared to its initial value with unperturbed water solution.

This Δn_eff_ was used to calculate the accumulated phase change over a length of one L_spp_, yielding a total phase change of 126 π/RIU/L_spp_ or 1.26 π/RIU/μm, when typical values for photonic waveguides, even those employing more sensitive slot based configurations compared to evanescent based alternatives, hardly exceeding 0.27 π/RIU/μm.

### Optical characterization of Al plasmonic stripes fabricated with optical lithography

**Figure 5:**
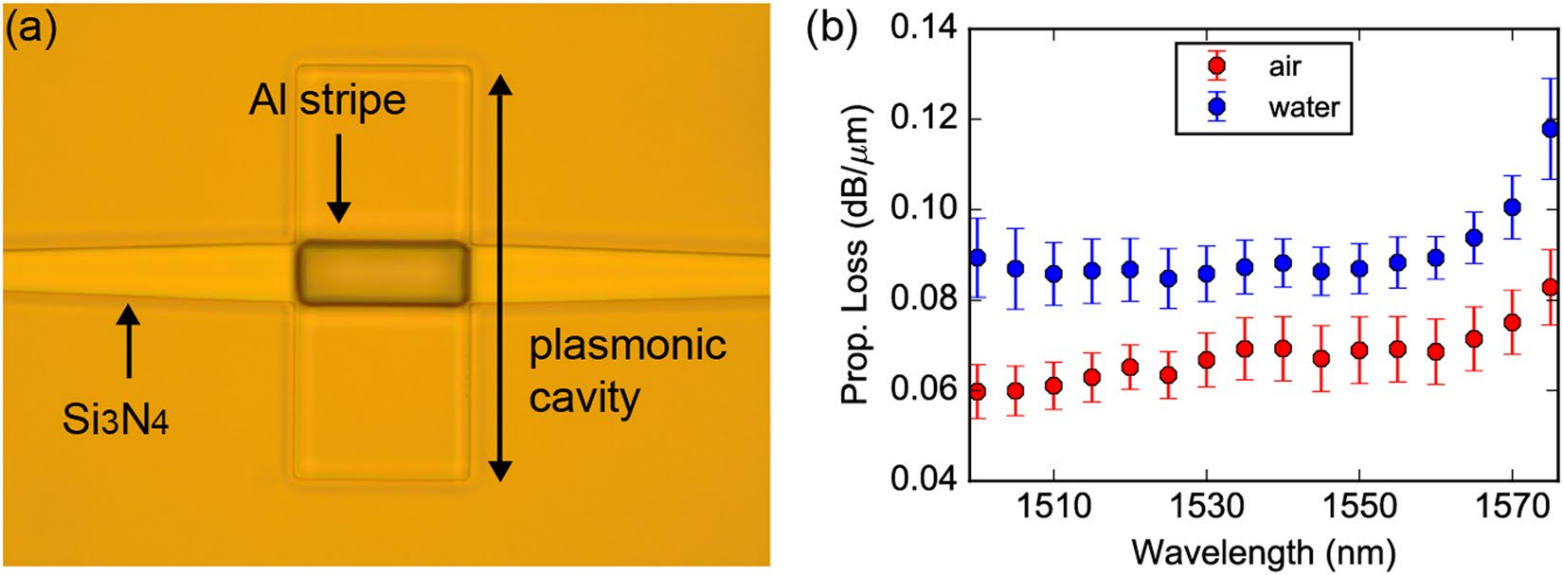
Fully CMOS manufactured Al plasmonic stripes co-integrated with Si_3_N_4_ photonics. (**a**) Optical microscope image showing in top-view a fully CMOS manufactured Al plasmonic stripe interface with Si_3_N_4_ waveguide, being recessed in a cavity. (**b**) Propagation loss of the plasmonic mode as function of wavelength in air and water.

After the successful demonstration of plasmonic propagation and true data transmission over Al plasmonic stripes fabricated with e-beam lithography, a second fabrication run was carried out employing optical lithography for the plasmonic stripe deposition and having both the photonic and plasmonic structures being fabricated within the same CMOS foundry of AMO. The plasmonic deposition rate was increased from 0.1 nm/s to 0.3 nm/s in an attempt to optimize the quality of Al stripes as suggested in the literature^19^. Figure 5(a) illustrates a microscope image of a fabricated plasmonic waveguide being deposited between the Si_3_N_4_ waveguides, while Fig. 5(b) shows the experimentally measured propagation loss of the plasmonic mode over wavelength in air and water. Experimental measurements have shown again successful plasmonic propagation, with plasmonic propagation losses of 0.068 dB/μm in air (L_spp-air_: 63.8 μm) and 0.087 dB/μm (L_spp-water_: 50 μm) in water, at 1.55 μm.

More specifically, the L_spp_ lengths at 1.55 μm have been increased by 27.6% and 39.6% in case of air and water, respectively, when compared to those obtained in the first fabrication run, presumably being the result of the increased deposition rate.

Unfortunately, increased Al-to-Si_3_N_4_ insertion losses of 17.6 dB in air and 15.5 dB in water, at 1.55 μm, have been measured, probably due to higher surface roughness at the vertical metallic edges of the interface sections that led to increased scattering losses during the mode transition. However, these higher interface losses proved not to be prohibitive in the evaluation of the propagation characteristics of the Al plasmonic waveguide.

## Discussion

The successful demonstration of a fully CMOS manufactured plasmo-photonic waveguide platform, where Al plasmonic waveguides have been co-integrated with low-loss Si_3_N_4,_ providing propagating SPP modes at 1.55 μm, paves the way towards future development of powerful plasmo-photonic devices, mainly addressing fundamental requirements imposed by biosensing and on-chip data transmission applications. In order to clarify that, we highlight those requirements and elaborate on the potential exploitation of our platform by comparing our results with those reported in the literature, thereby benchmarking the applicability of our platform in these two possible application scenarios. The experimentally evaluated performance characteristics of various SPP waveguides providing propagating plasmonic modes and employing either CMOS or noble based metals so far, are summarized in Table 1 following the terminology used in the literature^20^. The relevant performance characteristics of noble-metal-based SPP waveguides have been also incorporated in Table 1 to allow for a direct comparison between CMOS and noble-metal SPP waveguide structures. Au-based waveguides so far reported include Long-Range SPP waveguides that rely on symmetric plasmonic IMI waveguides. Such approach provides ultra-low loss plasmonic waveguides allowing for mm-scale L_spp_, however introducing strict design challenges and accurate fabrication control requirements.

**Table 1:** Experimental results for state-of-the-art CMOS compatible as well as noble-based SPP waveguides supporting propagating plasmonic modes.

Ref.Work	Plasmonic Waveguide	Metal	Co-integration with SOI*	Single Mode	Wavelength(λ)[μm]	SPP Prop. Loss (λ) [dB/ μm]		L_spp_ (λ) [μm]		Transmitted Data
						Air	Liquid	Air	Liquid	
**CMOS metals**										
^13^	LR-IMI	TiN	✗	✓	1.55	N.A.	0.0008	N.A.	5428.7	N.A.
^10^	DLSPP	TiN	✗	✓	1.55	0.43	N.A.	10.1	N.A.	N.A.
^10^	DLSPP	Cu	✗	✓	1.55	0.08	N.A.	54.3	N.A.	N.A.
^11^	Hybrid	Cu	✓	✓	1.55	0.14	N.A.	31.0	N.A.	N.A.
^12^	SR-IMI	Cu	✗	N.A.	0.63	✗	0.54–0.86	✗	8.0–5.0	N.A
^27^	MIM	Cu	✓	✓	1.55	0.37–0.78	N.A.	11.7–5.6	N.A.	N.A.
^27^	MIM	Cu	✓	✓	1.31	0.88	N.A.	4.9	N.A.	N.A.
^27^	MIM	Cu	✓	✓	1.06	3.2	N.A.	1.4	N.A.	N.A.
^27^	MIM	Cu	✓	✓	0.853	>5	N.A.	<0.9	N.A.	N.A.
^28^	MIM	Cu	✓	✓	1.55	0.17	N.A.	25.5	N.A.	N.A.
^10^	DLSPP	Al	✗	✓	1.55	0.15	N.A.	29.0	N.A.	N.A.
^16^	LR-DLSPP	Al	✓	✗	1.55	0.006	N.A.	723.8	N.A.	N.A.
^29^	CPP (V-Groove)	Al	✗	✗	0.514	1.03–1.13	N.A.	4.2–3.8	N.A.	N.A.
^15^	MIM	Al	✓	✓	1.55	1.01–1.56	N.A.	4.3–2.8	N.A.	N.A.
^22^	SR-IMI	Al	✓	✓	1.55	0.087	N.A	50	N.A	200 Gb/s (8 × 25 Gb/s)
**This Work**	**SR-IMI**	**Al**	✓	✓	**1.55**	**0.068**	**0.087**	**63.8**	**50**	**25 Gb/s**
**Noble metals**										
^30^	LR-IMI	Au	✗	✓	1.55	0.0034	N.A.	1277.3	N.A.	N.A.
^31^	LR-IMI	Au	✗	✓	1.55	0.0002	N.A.	21714.7	N.A.	40 Gb/s
^32^	LR-IMI	Au	✗	✓	1.55	0.00345	N.A.	1258.8	N.A.	196 Gb/s (4 × 49 Gb/s)
^33^	LR-IMI	Au	✗	✓	1.31	0.005	N.A.	868.58	N.A.	N.A.
^34, 35^	LR-DLSPP	Au	✗	✓	1.55	0.0086	N.A.	500	N.A.	10 Gb/s
^36^	LR-IMI	Au	✗	✓	1.31	0.012–0.017	N.A.	361.9–255.46	N.A.	N.A.
^37^	SR-IMI	Au	✓	✓	1.55	N.A	0.058	N.A.	75	N.A.
^9, 38^	DLSPP	Au	✓	✓	1.55	0.1	N.A.	43.42	N.A.	480 Gb/s (12 × 40 Gb/s)
^21^	Hybrid	Ag	✓	✓	1.55	N.A.	0.1	N.A.	40	N.A.

(*Either Si or SiN waveguides on Insulator), LR: Long-Range, SR: Sort-Range, IMI: Insulator-Metal-Insulator, DLSPP: Dielectric-Loaded Surface-Plasmon-Polariton, MIM: Metal-Insulator-Metal, CPP: Channel-Plasmon-Polariton).

In view of interferometric sensing applications, SPP modes that can be (i) fully exposed to liquid based claddings, (ii) being single-mode and transmitted over long propagation distances as well as (iii) interfaced to low-loss CMOS photonic waveguides, are imperatively needed to improve the sensitivity characteristics of biosensors and to allow for multi-channel biosensor setups. This challenging operational framework has been among the main targets of recent CMOS-metal plasmonic waveguide research but has still not been met by any of the experimental demonstrations reported so far. Only one of the Cu-based waveguide solutions has been shown to perform successfully in water-cladded conditions, counteracting surface oxidization via a successfully applied graphene protective layer but finally having rather pure plasmonic propagation lengths up to 8 μm^12^. On the other hand, TiN plasmonic waveguides have been shown to provide long-range propagation characteristics when cladded with a matching oil, yet disregarding compatibility with CMOS manufacturing standards due to the peculiar fabrication process that has been followed, as well as, the employed substrate material based on sapphire^13^. To this end, the Al plasmonic waveguides presented in this work as co-integrated elements on a Si_3_N_4_ waveguide platform, form currently the first demonstration of a plasmo-photonic waveguide platform suitable for CMOS-manufactured interferometric biosensor devices, demonstrating a propagation length of 50 μm in aqueous environment. Moreover, their Si_3_N_4_ co-integration allows for the utilization of all well-known passive functions offered by the photonic platform, including fiber-to-chip coupling via grating structures and on-chip SPP excitation. This negates the need for bulk optics towards exciting the SPP modes, allowing for significant reductions in the size of the entire device and contributing with new perspectives towards multi-channel interferometric plasmonic biosensor setups^3^. In this work, we demonstrated a plasmo-photonic waveguide that is intended for future use as the sensing transducer in interferometric devices, similar to that reported in^21^, however substituting the noble-metal based plasmonic waveguides with a fully CMOS compatible SPP waveguide.

In the context of on-chip data transmission applications, CMOS SPP waveguides having the prospect of direct co-integration with electronics, allow the simultaneous transmission of electrical and optical signals via the same waveguide structure naturally yielding to a stronger interaction between electrons and plasmons^5–8^. In addition, the proposed structure may be further developed as the means to replace energy efficient yet expensive to produce gold-based thermo-optic switches^7^ with low cost, CMOS compatible counterparts. In longer terms, the proposed work proves the feasibility of Al in integrated photonic circuits and opens new opportunities for mass manufacturing of plasmonic components with unmatched performances such as ultra-high speed plasmonic modulators^6^. A fundamental prerequisite towards this goal is the examination of the data carrying capabilities of the elementary waveguide structure as well as its evaluation in terms of BER measurements when being exposed to true data traffic conditions. As it can be seen in Table 1, this property has been mainly addressed so far in several SPP waveguide configurations using noble metals. Only our recently published work revealed the potential of CMOS plasmonic waveguides in Wavelength-Division-Multiplexed (WDM) data transmission using Al as the plasmonic material of choice, yet still employing a fabrication process that is not fully CMOS compatible and exhibiting higher plasmonic propagation losses than those reported in this work^22^. The CMOS compatible SPP waveguide presented in this work, can be used to replace the noble-metal based SPP counterparts that have been employed for the realization of thermo-optic switches, similar to that reported in^7^, yet in an CMOS compatible manner forming the base upon which further development of plasmo-photonic devices can be accomplished.

In conclusion, we have verified experimentally, the feasibility of co-integrating Al plasmonic waveguides with Si_3_N_4_ photonics employing low-cost and fully CMOS compliant material platforms and manufacturing methods. We opted for a silicon nitride based waveguide platform instead of silicon primarily due to the lower propagation losses and the higher immunity to fabrication imperfections originating from the smaller index contrast^23^. In addition, silicon nitride constitutes an attractive alternative compared to silicon contributing to the fabrication cost reduction since it is a deposited material. Propagating SPP modes over Al waveguides, exposed to water and air have been demonstrated at 1.55 μm with L_spp_ lengths of 50 and 65 μm, respectively, while air-cladded Al waveguides were also exposed to data traffic transmission experiments validating for the first time their high-quality signal integrity characteristics with 25 Gb/s optical data. The proposed integration scheme enables the seamless co-integration of low-cost and low-cost Si_3_N_4_ waveguides with Al based SPP waveguides in a CMOS fabrication line opening new perspectives towards CMOS plasmonic biosensing and on-chip data transmission applications. The Al based SPP waveguide reported in this work, exhibits the largest plasmonic propagation length among all the single mode Al based SPP waveguides so far.

## Methods

### Design and simulation of the butt-coupled Al-to-Si_3_N_4_ interfaces

We conducted 3D FDTD simulations calculating the insertion loss of the proposed interface at 1.55 μm for different LO and VO values. The quasi-TM mode of the tapered Si_3_N_4_ waveguide was launched during simulations towards the Al SPP waveguide and the power that is coupled only to its SPP mode was calculated, in this way determining the insertion loss of the proposed interface. For this purpose we utilized the built-in mode expansion monitor of *Lumerical Solutions*^20^ software that was settled 2 μm away from the front-end of the Al stripe (see Supplementary Fig. S1). The calculated insertion loss was not compensated for the propagation losses after a propagation distance of 2 μm. Simulations dictated plasmonic propagation length (L_spp_) of 64 and 104 μm at 1.55 μm in water and air, respectively. Norm plots of the Poynting vector (|Pz|) along the direction of propagation in a side-view, for different combinations of VO and LO values have shown clear SPP excitation on the Al stripes without any other mode being excited beneath them (see Supplementary Fig. S1). The refractive indices used in the simulations for the Si_3_N_4_, LTO, SiO_2_ and water at 1.55 μm were 1.996, 1.444, 1.444 and 1.311-0.0001348i, respectively^24,25^. During simulations we used the same refractive index value for both the LTO and the SiO_2_ layers. The complex refractive indices used for aluminum was based on experimentally verified data reported in the literature^10^.

### Fabrication of Si_3_N_4_ waveguides

The photonic waveguides have been fabricated using 150 mm silicon wafers where a 360 nm thick Si_3_N_4_ layer was deposited on top of a 2.2 μm thick thermal oxide in a Low-Pressure-Chemical-Vapor-Deposition (LPCVD) process. Marker layer and waveguides have been defined by optical projection lithography using an i-line stepper tool. Reactive-Ion-Etching (RIE) with CHF_3_ and He chemistry has been employed for the structure transfer. After the structure transfer, a 600 nm thick LPCVD SiO_2_ was deposited to form the cladding material of the photonic waveguide (LTO, Low-Temperature-Oxide) and annealed at 1000 °C fir several hours. The anneling process improved the optical properties of the oxide beign associated with the induced optical losses due its hygroscopic properties. The plasmonic cavity has been defined also by the same stepper tool and etched via RIE through LTO, Si_3_N_4_ and 400 nm thermal oxide. The resulting cavity depth was 1.36 µm. Schematics illustrating the employed fabrication process flow are provided in the Supplementary Fig. S2.

### Fabrication of Al plasmonic waveguides using e-beam lithography

Plasmonic structures have been defined firstly by depositing Al following a lift-off process and e-beam lithography, thermal evaporation of aluminum and lift-off. The e-beam tool was operated at 20 kV acceleration voltage on a All resist AR-P 679 PMMA resist film with a thickness of 600 nm which has been coated on the samples that contained the cavities at 1500 rpm. The alignment markers that have been already defined in the Si_3_N_4_ layer and cladded by LTO augmented the alignment process of the e-beam tool for the subsequent lithography step. Afterwards, the resist was developed in the AR 600-56 developer for one minute followed by rinsing in AR 600-60 for 30 seconds and drying with nitrogen. A descumming step followed for 1.5 minute in an oxygen-plasma cleaner prior the evaporation of the metal. Al has been evaporated using a Plassys MEB 400 e-gun evaporator with 99.999% pure aluminum pellets obtained from Neyco and being loaded in vitrified graphite crucible. The evaporation rate was 0.1 nm/s targeting an Al thickness of 80 nm. The thickness of the Al was monitored with a quartz crystal microbalance during the evaporation process. An AR 600-71 remover bath operated at 50 °C was used for the lift-off process. At the end the chips have been rinsed in acetone and isopropanol and blown dry with nitrogen. Schematics illustrating the employed fabrication process are provided in the Supplementary Fig. S3.

### Fabrication of Al plasmonic waveguides using optical lithography

In this fabrication run, all process steps have been accomplished within the same CMOS foundry. The crucial part during the fabrication of Al plasmonic stripes is to ensure that no Al residues remain on the edges of the photonic waveguide. Only a lift-off process enables clean side walls of the photonic-plasmonic interface. Using a lift-off process, a resist layer covers the whole wafer only leaving stripes where the plasmonic structures have to be defined uncovered. Afterwards, aluminum is deposited on top. A bath in solvents removes the resist layer with the unwanted aluminum on top. Only the aluminum remains, which is placed directly on top of the surface without resist beneath. The step like profile with the 1.36 µm high side walls makes the lift-off challenging because of a high probability of re-depositions of the lifted material. For defining the resist structures, a thick layer of negative tone image reversal resist was used with an additional lift-off resist (LOR) layer which is highly affected by the developer to create a sufficient undercut. Once again an i-line stepper system was used, this time to expose the lift-off resist structures. After resist development, 80 nm of aluminum were deposited using electron beam evaporation with a deposition rate of 0.3 nm/s in order to provide minimum sidewall coverage of the resist layer. Dimethyl sulfoxide (DMSO) was used to minimize re-deposition of aluminum. DMSO was removed by an isopropyl alcohol rinse with subsequent nitrogen drying step. Schematics illustrating the fabrication process flow that was followed are provided in the Supplementary Fig. S4.

### Optical characterization

We performed broadband optical characterizations by sweeping the wavelength of a Tunable-Laser-Source (ANDO AQ4321) from 1.5 to 1.575 μm, using PM fibers at the input of the chip to ensure that TM-polarized light is coupled into the Si_3_N_4_ waveguides. The FtF loss budget of the characterized Al plasmonic stripes as a function of wavelength is shown in Supplementary Fig. S5. The propagation losses of the SPP mode over wavelength have been acquired after applying a linear fitting process to the experimentally obtained dataset. The least-squares method has been followed during the linear fitting process. The insertion loss of the proposed Al-to-Si_3_N_4_ interface, per transition, has been determined after subtracting the FtF losses attributed to the reference Si_3_N_4_ waveguide structure from the intercept values, which have been obtained by the linear fitting process, and the resulting loss value was divided by a factor of two.

### BER measurements

BER measurements have been carried out utilizing a BER tester (ADSANTEC ED45 2CH). The BER curves have been obtained after measuring BER values for increasing mean optical power levels of the optical signal that reached the photo-receiver. Those curves have been fitted following methods being reported in the literature^26^. The reference waveguide structure was used during the Back-to-Back (BtB) measurements, exhibiting an optical loss of 26.5 dB which is attributed to the insertion loss of two Si_3_N_4_ GCs (26 dB) as well as the propagation loss of the Si_3_N_4_ waveguide with a length of 0.88 cm. External optical attenuation of 9 ± 0.5 dB was applied to the link using a fiber pigtailed attenuator to incorporate the insertion loss of the two Al-to-Si_3_N_4_ interfaces.

## Electronic supplementary material

Supplementary Information
