# Supplementary material for: Aluminum plasmonic waveguides co-integrated with Si3N4 photonics using CMOS processes
Source: Sci Rep. 2018 Sep 6;8:13380. doi: 10.1038/s41598-018-31736-4 (PMC6127305; doi:10.1038/s41598-018-31736-4)
Supplement: Supplementary file 1 — Supplementary Information [file 41598_2018_31736_MOESM1_ESM.pdf]

Supplementary Information for

Aluminum plasmonic waveguides co-integrated with Si_3N_4 photonics using CMOS processes

George Dabos[1,*], Athanasios Manolis[1], Dimitris Tsiokos[1], Dimitra Ketzaki[1], Evangelia Chatzianagnostou[1], Laurent Markey[2], Dmitrii Rusakov[2], Jean-Claude Weeber[2], Alain Dereux[2], Anna-Lena Giesecke[3], Caroline Porschatis[3], Thorsten Wahlbrink[3], Bartos Chmielak[3] and Nikos Pleros[1]
.
[1]Department of Informatics, Center for Interdisciplinary Research and Innovation, Aristotle University of Thessaloniki, 10th Km Thessalonikis-Thermis Av., 57001, Greece
[2] Laboratoire Interdisciplinaire Carnot de Bourgogne, UMR 6303 CNRS-Université de Bourgogne
[3]AMO GmbH, Advanced Microelectronic Center Aachen (AMICA), Otto-Blumenthal-Strasse 25, 52074, Aachen, Germany
*ntamposg@csd.auth.gr

Design and simulation of the butt-coupled Al-to-Si_3N_4 interface

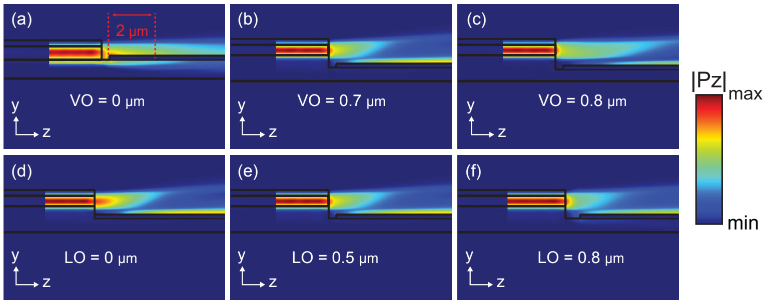

Supplementary Figure S1 Simulated norm of the Poynting vector $|P_z|$ along the direction of propagation for varied vertical (VO) and longitudinal (LO) offsets. (a-c) $|P_z|$ along the direction of propagation for vertical (VO) offsets of 0, 0.7 and 0.8 µm, respectively. (d-f) $|P_z|$ along the direction of propagation for longitudinal (LO) offsets of 0, 0.5 and 0.8 µm, respectively.

Samples fabrication
Fabrication of Si_3N_4 waveguides.

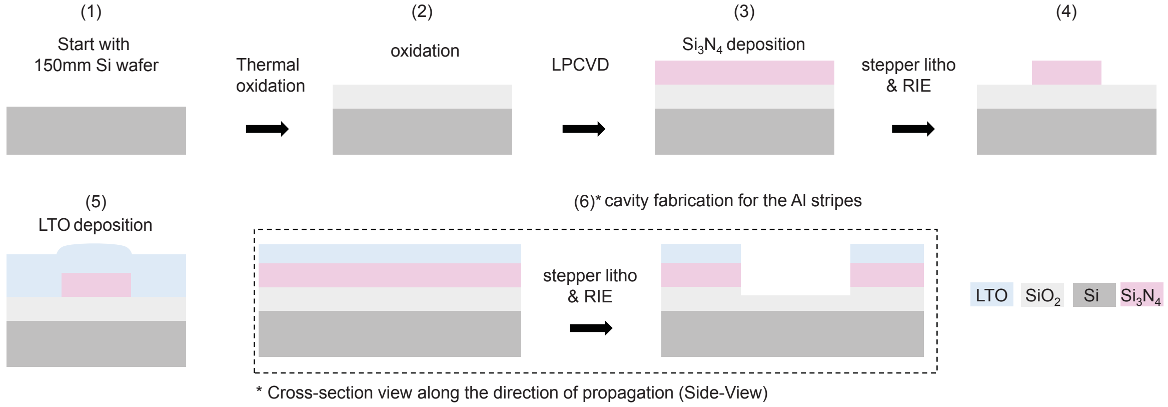

Supplementary Figure S2 Fabrication process flow of the Si_3N_4 waveguides and the cavity for Al stripes using optical projection lithography and an i-line stepper tool.

Fabrication of Al plasmonic waveguides using e-beam lithography.

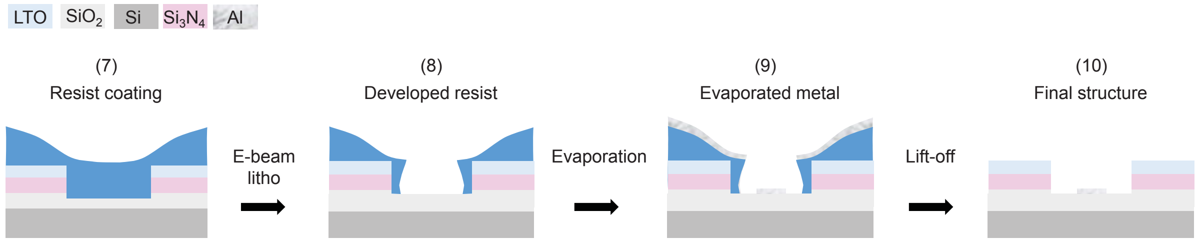

Supplementary Figure S3 Fabrication process flow for the Al plasmonic stipes using e-beam lithography and an evaporation speed of 0.1 nm/s.

Fabrication of Al plasmonic waveguides using optical lithography.

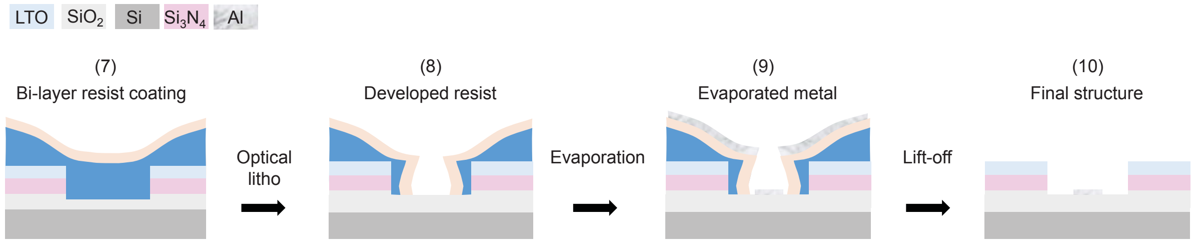

Supplementary Figure S4 Fabrication process flow of the Al plasmonic stipes using optical projection lithography and an in-line stepper tool. The evaporation speed of Al was 0.3 nm/s.

Optical characterization

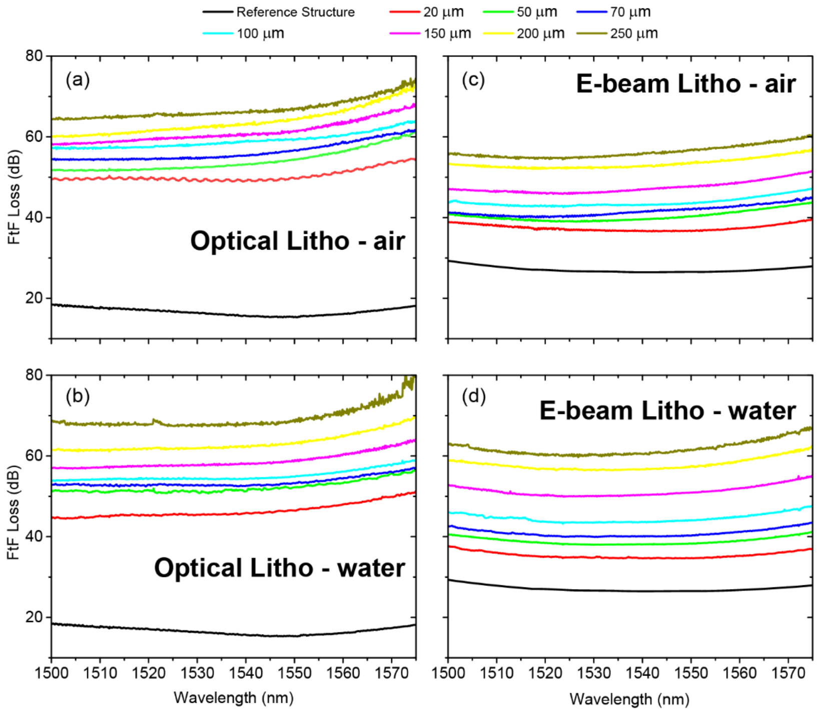

Supplementary Figure S5 Fiber-to-fiber loss measurements for the tested structures and Al plasmonic waveguides with varied length that have been fabricated with (a,b) optical and (c,d) E-beam lithography, respectively.
